# Under-recognized Etiology of Altered Mental Status in a Patient with Alcoholism

**DOI:** 10.5811/cpcem.2018.8.39266

**Published:** 2018-10-16

**Authors:** James D. Maloy, Ronny M. Otero

**Affiliations:** Beaumont Health System, Department of Emergency Medicine, Royal Oak, Michigan

## Abstract

Wernicke’s encephalopathy is an important condition for the emergency physician (EP) to consider in patients at risk for malnutrition. A 60-year-old man with history of alcoholism presented with word-finding difficulties, dysmetria, ataxia, and personality changes. After treatment with high-dose thiamine, his neurological status returned to his baseline. Although EPs routinely prescribe thiamine for patients with alcoholism, the common initial dose of 100 mg per day is likely subtherapeutic, and the population of patients at risk for malnutrition is much broader than only those with alcoholism, and includes those with cancer, anorexia nervosa, hyperemesis gravidarum, and others. EPs must be aware of this low-cost, readily available prophylaxis to prevent long-term neurological morbidity.

## INTRODUCTION

We report a case of Wernicke’s encephalopathy that presented with ataxia and confusion but without ocular involvement. This departure from the “classic” presentation of ataxia, confusion, and opthalmoplegia is common and likely under-recognized.^2^ We describe this patient’s seemingly unusual presentation and his subsequent improvement with high-dose thiamine. We also discuss evidence that this disorder is common not only in those with alcoholism but in other populations at risk for malnutrition.^2^ Prescription of high-dose thiamine to such patients represents an opportunity for emergency physicians (EP) to pursue a low-cost, highly effective public health intervention that prevents long-term neurological disability.

## CASE REPORT

A 60-year-old man with known history of alcoholism and mild vascular dementia presented to the emergency department (ED) due to a change in mental status. The patient’s wife reported that for three weeks the patient had subtle personality changes, word-finding difficulties, gait worsening from prior independent ambulation to requiring a walker. Furthermore, the patient had difficulty standing up from the toilet earlier that same day, falling back onto the toilet seat. He did not hit his head or lose consciousness. The patient denied any focal weakness or numbness. He’d had no fevers or chills, headache, change in vision, respiratory symptoms, or urinary symptoms. The patient did endorse drinking daily; he was unable to quantify his alcohol intake, but estimated he had between 5–10 drinks daily. He did drink the day of presentation to the ED.

On physical exam, vitals were stable. The patient was a talkative, obese man with word-finding difficulties. His cranial nerves were normal; in particular, there was no abnormality in ocular movements and no nystagmus. There was no focal weakness or numbness. Finger-nose-finger testing revealed symmetric bilateral dysmetria. The patient declined evaluation of his postural stability and gait. Laboratory evaluation was largely unremarkable, and head computed tomography demonstrated chronic ischemic changes without mass lesion or bleed (Image).

In the ED, the patient received 500 mg of intravenous (IV) thiamine. He was admitted for altered mental status concerning for Wernicke’s encephalopathy. He received 100 mg of IV thiamine daily, and by day three his mentation improved and his dysmetria resolved. He continued to require a walker to ambulate. The patient resolved to quit drinking. He was discharged from the hospital on a regimen of 100 mg of oral thiamine daily.

The patient followed up with a neurologist approximately one month after hospital discharge. At that visit, the neurologist noted that the patient’s memory and cognitive function had improved. He had mild gait instability and occasional falls, but this had improved as well. Neurologic exam was otherwise normal, including normal finger-nose-finger, heel-to-shin, and rapid repetitive and rapid alternating movements. The neurologist agreed with the clinical diagnosis of Wernicke’s encephalopathy, but the patient refused to undergo magnetic resonance imaging (MRI) due to his claustrophobia.

## DISCUSSION

Wernicke’s encephalopathy is a neurologic disorder caused by lack of thiamine (vitamin B1) and most often affects patients with chronic alcoholism, although it also disproportionately affects patients with acquired immunodeficiency syndrome, hyperemesis gravidarum, malignancies, post-bone marrow transplants, post-bariatric surgery, as well as patients with eating disorders and others.^1^,^2^ If untreated, the disease may progress to Korsakoff’s syndrome, which is characterized by chronic, largely irreversible, primarily anterograde amnesia.^3^ Treatment with thiamine can prevent progression to Korsakoff’s syndrome. Although some patients may demonstrate radiographic evidence of the disease, most notably contrast enhancement of the mammillary bodies and thalami on MRI, these features are poorly sensitive.^2^,^4^,^5^

**CPC-EM Capsule**

What do we already know about this clinical entity?

Wernicke’s encephalopathy, classically characterized by confusion, ataxia, and ophthalmoplegia, is a complication of malnutrition and is treated with thiamine.

What makes this presentation of disease reportable?

Here is reported a case of Wernicke’s presenting only with confusion and ataxia, without ocular involvement.

What is the major learning point?

Wernicke’s must be suspected in any patient at risk of malnutrition, even without the classic triad, and must be treated with high-dose thiamine.

How might this improve emergency medicine practice?

Emergency physicians must recognize patients at risk for malnutrition and administer higher-than-usual doses of thiamine to prevent permanent neurologic disability.

Although red blood cell transketolase levels can be low in thiamine deficiency and serum thiamine levels can be measured, laboratory studies are seldom helpful in practice. Therefore, diagnosis is made clinically. The classic triad includes opthalmoplegia, ataxia, and confusion. However, few patients clearly demonstrate all three signs (fewer than 16% by one report) and this schema does not depict the true breadth of symptomatology possible in patients with Wernicke’s, which more broadly includes any eye movement abnormality, any change in gait, or any change in mental status.^2^ Wernicke’s encephalopathy is likely under-recognized and undertreated: the prevalence of Wernicke-Korsakoff syndrome (WKS) in alcoholics is as high as 12.5%, and autopsies have demonstrated that over 80% of true cases are not diagnosed during life.^2^ Furthermore, the appropriate dosing strategy for thiamine is not well-established. The frequently encountered dose of 100 mg IV daily is common for historical reasons and is not evidence-based. A 2013 Cochrane Review concluded that “evidence […] is insufficient to guide clinicians in determining dose, frequency, route or duration of thiamine treatment for prophylaxis against or treatment of WKS.”^6^ Thiamine is largely benign and well tolerated, and inadequate treatment is associated with significant, permanent neurocognitive disability. Based on this, one study published in *Annals of Emergency Medicine* recommends, “For patients for whom there is low suspicion for disease or for those simply requiring prophylaxis, a minimum of 100 mg should be administered intravenously. For those with confirmed or highly suspected disease and those who have ‘failed’ the 100-mg regimen, we recommend a dosage upwards of 500 mg intravenously.”^4^ In summary, the differential diagnosis of altered mental status in a patient with alcoholism must always include Wernicke’s encephalopathy, even without ocular findings or objective malnutrition, and one should have a low threshold to treat with high-dose thiamine to prevent progression to permanent neurocognitive disability.

## CONCLUSION

The differential diagnosis of altered mental status in a patient with alcoholism is broad and varied, but patients with Wernicke’s encephalopathy secondary to thiamine deficiency are likely under-recognized and undertreated, which leads to significant, permanent, avoidable neurocognitive disability. EPs should maintain a high index of suspicion for Wernicke’s in any patient with alcoholism or any other disorder predisposing to malnutrition who presents with altered mental status, even without the classic signs, and have a low threshold to treat with higher-than-usual doses of thiamine.

Documented patient informed consent and/or Institutional Review Board approval has been obtained and filed for publication of this case report.

## Figures and Tables

**Image:**
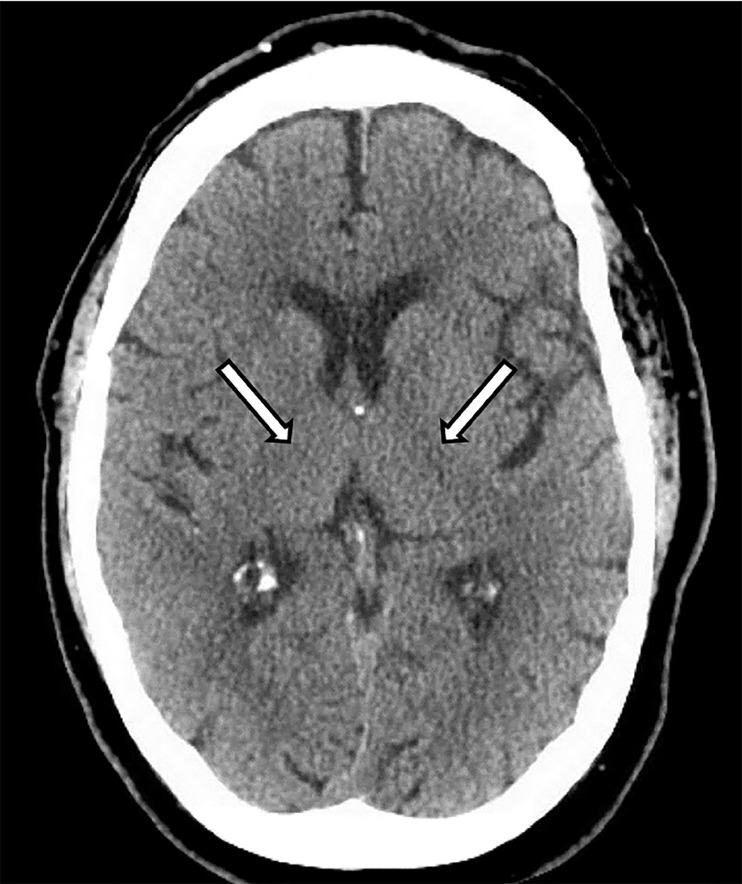
Computed tomography (CT) brain of patient with Wernicke encephalopathy (WE). The utility of CT in WE is primary to rule out alternative diagnoses. Here, there is periventricular hypoattenuation of the white matter (arrows) without evidence of any other alternative diagnoses. This is nonspecific but consistent with chronic, small-vessel ischemic disease, which is consistent with his baseline mild vascular dementia but is insufficient as an explanation for his more acute neurological deficits. Magnetic resonance imaging would demonstrate findings specific for WE. Notably, this altered patient had difficulty remaining still for the examination, giving rise to significant motion artifact.
